# 112 Developing Outpatient Registry to Capture Data Post Hospitalization

**DOI:** 10.1093/jbcr/irac012.115

**Published:** 2022-03-23

**Authors:** Bethany A Schmoker, Shane Urban, Arek J Wiktor

**Affiliations:** UC Health University of Colorado Hospital, Strasburg, Colorado; University of Colorado Hospital, Denver, Colorado; University of Colorado Burn Center, Aurora, Colorado

## Abstract

**Introduction:**

Per the 2019 ABA reverification requirements, a burn center must see >75% of all inpatients (IP) who require an outpatient (OP) follow-up after discharge. In prior years, we utilized the inpatient registry and built a report to track patient follow-up. With the report, we were able to compare the number of Burn Clinic return patients against admissions to get the percentage. This process required hours of focused effort. We sought to optimize the process for determining IP follow-up at our ABA verified burn center. In addition, we hoped to better quantify the efficacy of our OP clinic.

**Methods:**

An OP registry was developed in December 2019 utilizing an automated report from our electronic medical record (EMR) and imported into a custom built, secure, web-based software platform designed to support data capture for research studies. Employing various automation techniques, we were able to eliminate the need for manual abstraction by our burn registry team. Metrics tracked in the OP registry included: type of patient visit (New Patient, Return Patient, and Telehealth), diagnoses, zip-codes of patient residence, payer methods, and total number of clinic encounters per year. We collected data from January 2020 through the present, with 2020 being the first full year in the OP registry. The initial effort required to design, automate, and import data was approximately 18 hours. The report import takes approximately 5 minutes.

**Results:**

The OP registry has given us the ability to create a multitude of graphs from the OP clinic data, like the one shown. During the review period our OP clinic saw patients from 19 different US states, encompassing 2,710 total OP visits. The median number of monthly OP clinic visits was 235 [IQR 210-246], see graph 1. The median number of clinic visits per patient was 2 [IQR 1-4]. The majority of clinic visits were return patients (55%, n = 1595), new patients (31%, n = 914), and telehealth visits (14%, n = 399). Finally, our analysis of the OP Clinic Registry demonstrated that we saw 82% (309/374) of inpatients that required follow-up care, exceeding the expected 75% by the ABA.

**Conclusions:**

The creation of an automated OP registry can assist the tracking of discharged patients and reduce the amount of effort needed to track ABA required metrics. In addition, this OP registry can be expanded to track both IP and OP outcomes. This is crucial for quality improvement for the burn program as a whole.

